# A Case Report on Longitudinal Collection of Tumour Biopsies for Gene Expression-Based Tumour Microenvironment Analysis from Pancreatic Cancer Patients Treated with Endoscopic Ultrasound Guided Radiofrequency Ablation

**DOI:** 10.3390/curroncol29100531

**Published:** 2022-09-21

**Authors:** Patrick V. Lawrence, Krisha Desai, Christopher Wadsworth, Nagina Mangal, Hemant M. Kocher, Nagy Habib, Anguraj Sadanandam, Mikael H. Sodergren

**Affiliations:** 1 Division of Molecular Pathology, The Institute of Cancer Research, London SM2 5NG, UK; 2 Department of Surgery and Cancer, Imperial College London, London W12 0HS, UK; 3 Centre for Tumour Biology, Barts Cancer Institute—A CRUK Centre of Excellence, Queen Mary University of London, London EC1M 5PZ, UK; 4 Barts and the London HPB Centre, The Royal London Hospital, Barts Health NHS Trust, London EC1M 5PZ, UK; 5 Barts Pancreas Tissue Bank, Barts Cancer Institute—A CRUK Centre of Excellence, Queen Mary University London, London EC1M 5PZ, UK; 6 Centre for Global Oncology, Division of Molecular Pathology, The Institute of Cancer Research, London SM2 5NG, UK

**Keywords:** fine-needle aspiration biopsy, pancreatic ductal adenocarcinoma, tumour microenvironment, immune checkpoint genes, radiofrequency ablation

## Abstract

Background: Most patients with pancreatic ductal adenocarcinoma (PDAC) are metastatic at presentation with dismal prognosis warranting improved systemic therapy options. Longitudinal sampling for the assessment of treatment response poses a challenge for validating novel therapies. In this case study, we evaluate the feasibility of collecting endoscopic ultrasound (EUS)-guided longitudinal fine-needle aspiration biopsies (FNABs) from two PDAC patients and conduct gene expression studies associated with tumour microenvironment changes associated with radiofrequency ablation (RFA). Methods: EUS-guided serial/longitudinal FNABs of tumour were collected before and after treatment from two stage III inoperable gemcitabine-treated PDAC patients treated with targeted RFA three times. Biopsies were analysed using a custom NanoString panel (144 genes) consisting of cancer and cancer-associated fibroblast (CAFs) subtypes and immune changes. CAF culture was established from one FNAB and characterised by immunofluorescence and immunoblotting. Results: Two-course RFA led to the upregulation of the CD1E gene (involved in antigen presentation) in both patients 1 and 2 (4.5 and 3.9-fold changes) compared to baseline. Patient 1 showed increased T cell genes (CD4—8.7-fold change, CD8—35.7-fold change), cytolytic function (6.4-fold change) and inflammatory response (8-fold change). A greater than 2-fold upregulation of immune checkpoint genes was observed post-second RFA in both patients. Further, two-course RFA led to increased PDGFRα (4.5-fold change) and CAF subtypes B and C genes in patient 1 and subtypes A, B and D genes in patient 2. Patient 2-derived CAFs post-first RFA showed expression of PDGFRα, POSTN and MYH11 proteins. Finally, RFA led to the downregulation of classical PDAC subtype-specific genes in both patients. Conclusions: This case study suggests longitudinal EUS-FNAB as a potential resource to study tumour and microenvironmental changes associated with RFA treatment. A large sample size is required in the future to assess the efficacy and safety of the treatment and perform comprehensive statistical analysis of EUS-RFA-based molecular changes in PDAC.

## 1. Introduction

Pancreatic ductal adenocarcinoma (PDAC) is the third most common cause of cancer death after colorectal and lung cancer [1], with a five-year survival rate of <5% despite significant advances in cancer management for other solid organ malignancies over the last few decades. At presentation, only 5–25% of patients are eligible for radical curative surgery, and, even in these patients, only 30% of these patients will survive five years [2]. At diagnosis, ~35% of patients have locally advanced, unresectable, stage 3 disease, usually due to significant encasement of the superior mesenteric vein/portal vein or involvement of the superior mesenteric or hepatic arteries. The prognosis for this group of patients is extremely poor, with a median survival of 12–14 months on chemotherapy. While a small proportion of these patients respond well to chemotherapy and can subsequently undergo surgery, palliative chemotherapy remains the only treatment option in most patients [3,4,5].

Radiofrequency ablation (RFA) describes the destruction of the tumour using heat generated by a high frequency alternating current applied through an electrode tip. The tissue is heated to 60 °C, resulting in coagulative tissue necrosis at the centre of the ablation zone. RFA is used to treat a few solid organ malignancies and is routinely used in primary hepatocellular carcinoma (HCC) [6,7]. However, the complete eradication of stage 3 PDAC is not usually possible with RFA due to tumour proximity to major vascular structures and the duodenum and a consequent risk of injury. Nevertheless, RFA of inoperable stage 3 PDAC at laparotomy has been found to be safe [8]. Indeed, a large series examining the procedure reported a median overall survival (OS) and disease-specific survival (DSS) of 20 and 23 months, respectively, with RFA-related morbidity of 15% and overall mortality of 3% [9]. A separate study has revealed a median survival of 12–14 months for same-stage patients on standard chemotherapy [3]. 

Following RFA, the localised necrosis of the tumour initiates a cascade of events, including a release of proinflammatory signals, cellular debris representing a source of tumour antigens and a host adaptive immune response against the tumour [10]. RFA appears to induce both innate and adaptive immune responses against the tumour through an effective infiltration of dendritic cells, boosted antigen presentation, and an intensified T cell response [11,12,13,14,15]. Recent studies have suggested that cancer-associated fibroblasts (CAFs), which are central components of the desmoplastic stroma found in most PDACs, can positively or negatively impact anti-tumour immune responses [16,17,18,19]. We and others have recently reported heterogeneity in CAFs and identified different subtypes (subtypes A–D or inflammatory (i)CAF/myofibroblast (my)CAF/antigen presenting (ap)CAF) with distinct associations with immune cells [18,20]. The spatial distribution of CAFs may most likely drive the immune exclusion phenotype, which is a defining feature of PDAC [21,22]. Moreover, cancer subtypes (classical, quasi-mesenchymal (QM) and exocrine-like) and similar subtypes of PDAC have been shown to be associated with immune/stromal changes and patient prognosis [18,23,24,25]. The recent discovery of tertiary lymphoid structures elucidating the specific role of B cells [26,27], T cells and NK cells [28] within the PDAC tumour microenvironment (TME) warrant a comprehensive understanding of these cell types, especially when assessing anti-tumour responses associated with RFA in which the immune system plays a critical modulatory role.

There have been no previous reports of longitudinal tumour sampling in PDAC patients when treated with radiofrequency ablation. It is also unknown whether minimally invasive tissue sampling through endoscopic ultrasound (EUS)-guided fine-needle aspiration biopsy (FNAB) is adequate for sampling tissues subjected to RFA for cellular and molecular analyses. This proof-of-concept study therefore aimed to assess the feasibility of longitudinally collecting FNABs for studying tumour microenvironmental changes associated with RFA in PDAC.

## 2. Methods

### 2.1. Study Design

Two patients were recruited into the pilot phase of the ARDEO trial (a phase II prospective randomised clinical study of endoscopic ultrasound guided radiofrequency ablation for inoperable pancreatic ductal adenocarcinoma; UK REC reference: 18/SW/0103). They underwent 3 × 28 days of gemcitabine treatment, received 3 endoscopic ultrasound radiofrequency ablation procedures on day 20 of each chemotherapy cycle, and sequential EUS FNABs were taken during each procedure prior to RFA treatment. Written informed consent was taken from the patients before enrolling into the study. PDAC patients were recruited over 2 months at the Hammersmith Hospital, Du Cane Road, London. All EUS-RFA procedures were uneventful with no observed clinical complications in these two patients. However, this study is not to assess the efficacy and safety of RFA treatment. Hence, a large study in the future is required to perform this assessment.

### 2.2. Endoscopic-Ultrasound Guided Radiofrequency Ablation

Following EUS examination of the pancreas and primary tumour site, an FNAB of the tumour was taken and then targeted RF delivered using 10 Watts for 2 min per application using the Habib EUS RFA (Boston Scientific, Marlborough, MA, USA) [29]. For the second and third EUS examinations, the ablation zone was examined, and a fine-needle biopsy was taken prior to RFA. Two FNABs per time point were collected using a 22G needle, one dropped in transport media for culture and another snap frozen in liquid nitrogen and transported on dry ice. Routine biochemistry tests including bilirubin and CA19-9 were analysed at Imperial College Healthcare NHS Trust laboratory.

### 2.3. Nucleic Acid Extractions from FNABs

Flash-frozen FNABs were homogenised in Precellys beaded tubes (Bertin Technologies^TM^, Montigny-le-Bretonneux, Saint-Quentin-en-Yvelines, France) containing 700 μL of lysis buffer using a tissue homogeniser (Bertin Technologies^TM^, Montigny-le-Bretonneux, Saint-Quentin-en-Yvelines, France). RNA from tumour lysates was extracted using AllPrep DNA/RNA/miRNA Universal Kit (Qiagen^TM^, Manchester, UK) according to the manufacturer’s instructions. RNA yield was estimated by Nanodrop 2000 (Thermo Fisher Scientific^TM^, Waltham, MA, USA).

### 2.4. Transcriptomic profiling on NanoString^TM^ nCounter

An amount of 100 ng of total mRNA was used to run a custom designed human gene panel encompassing cancer, immune and cancer-associated fibroblast genes along with housekeeping reference genes for data normalization (144 genes). Tumour samples were run on the nCounter^®^ Max analysis system (NanoString Technologies, Seattle, WA, USA) as per the protocol previously published by us [18,30]. Data were assessed for quality, normalised to six housekeeping genes (*AMMECR1L*, *DHX16*, *DNAJC14*, *PRPF38A*, *TMUB2* and *ZNF384*) and log_2_-transformed using nSolver 4.0 software. Gene expression values were plotted using GraphPad Prism Version 8.0 (for Windows, GraphPad Software, La Jolla, CA, USA.

### 2.5. Gene Scores and Subtypes

Gene scores for cancer subtypes, T cell, B cell, cytolytic function and inflammation were obtained by averaging the expression of genes specific to the respective subtype, cell types or immune function [31,32,33,34,35,36]. A list of subtype- and cell-type-specific genes used here to arrive at the scores can be found in Table S1. CAF subtype scores were derived by averaging the expression of the top differentially expressed genes representing pCAF subtypes [18] for each sample. PDAC subtypes [23] were predicted by the nearest template prediction method (NTP) [37] using subtype-specific genes identified previously [23].

### 2.6. Isolation of Patient-Derived Cancer-Associated Fibroblasts from EUS-FNABs

Cancer-associated fibroblasts from fresh FNABs were isolated by outgrowth method [38,39]. Cells were screened for mycoplasma by polymerase chain reaction and found to be negative (Figure S1). DNA for STR profiling was sent to Eurofins Genomics (Wolverhampton, UK), Figure S2. See Supplementary Methods for detailed protocol.

### 2.7. Statistics

To assess RFA-mediated transcriptome modulation in serial FNABs, log_2_ transformed gene expression values at T2 (after the 1st RFA) and T3 (after the 2nd RFA) were subtracted from T1 (baseline) and denoted as *difference*. Then, 2^ (*difference*) was performed to calculate fold change in gene expression over the course of RFA treatment.

## 3. Results

### 3.1. Feasibility of Collecting Longitudinal EUS FNABS to Study Immune Microenvironment of RFA-Treated Patients

**Figure 1:**
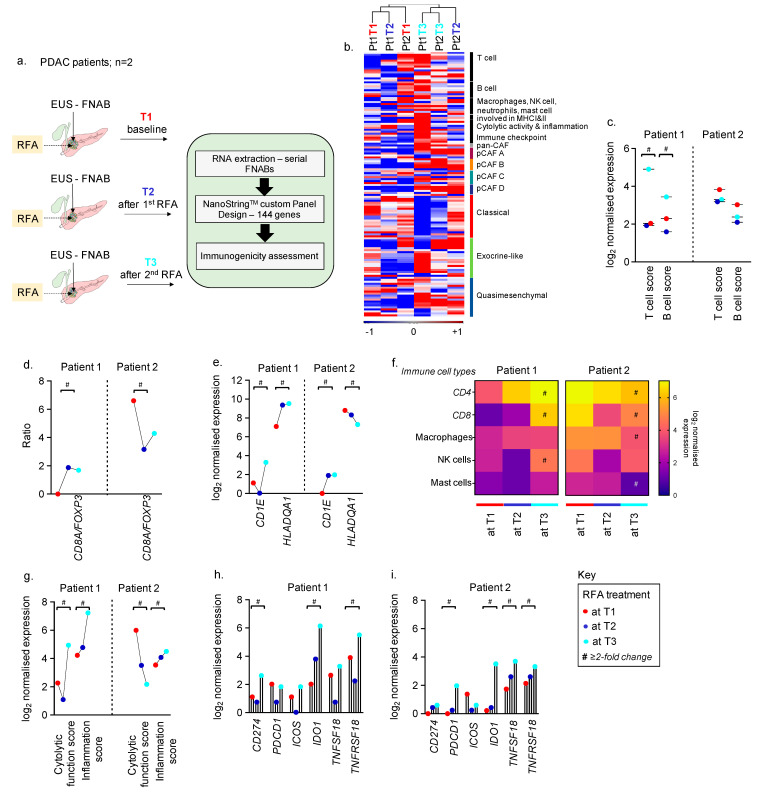
Longitudinal FNAB-based immunogenicity in PDAC patients treated with RFA. (**a**) Schematic illustration of longitudinal FNABs and RFA courses along with approaches used in this study. (**b**) Heatmap depicting distribution of genes across samples and grouped by unsupervised hierarchical clustering. Scale bar indicates log_2_ normalised expression ranging between −1 and +1. Colour bars represent appropriate CAF and PDAC subtype-specific colours. (**c**) Change in T and B cell scores at T1, T2 and T3 in patient 1 and 2, respectively. (**d**) Ratio of *CD8A/FOXP3* in patient 1 and 2. (**e**) Change in expression of genes participating in antigen presentation via MHC-I and II at T1, T2 and T3 in patient 1 and 2, respectively. (**f**) Heatmap representing RFA-mediated regulation of genes associated with different immune cell types; single scale bar for both patients depicting log_2_ normalised expression. (**g**) Change in tumour-immune response in pre- and post-RFA-treated FNABs measured as cytolytic function and inflammatory score. (**h**,**i**) Upregulation of immune checkpoint genes as a result of RFA in patient 1 and 2, respectively. # represents greater than or equal to two-fold change in gene expression between T1 and T3. Colour key represents T1 in red, T2 in blue, and T3 in cyan.

To assess the utility of longitudinally collected FNABs and RFA-mediated modulation of the tumour microenvironment (TME), we performed transcriptomic profiling of FNABs collected longitudinally over three courses of RFA for the two patients enrolled in our proof-of-principle study. Serial EUS-RFA treatments were well-tolerated with no complications. Both patients had stable disease immediately after EUS-RFA, but with different survival outcomes. A schematic representation of sample collection and approaches is outlined in Figure 1a. A custom NanoString panel was designed to include genes for PDAC and CAF subtypes identified previously [18,23], along with markers to assess changes in the immune-specific TME as a result of RFA treatment. Initial unsupervised hierarchical clustering of the six tumour samples grouped T3 FNABs (after the second RFA) of both the patients together and showed enrichment of T cell markers, immune checkpoint genes and increased fibrosis observed as upregulated CAF markers (Figure 1b).

We first assessed RFA-induced adaptive immune gene changes in longitudinally collected biopsies. A high T (7.3-fold) and B (2.2-fold) cell score at T3 in patient 1 compared to baseline and T2 was observed. There was no change in T and B cell scores in patient 2 at T2 and T3 compared to baseline (Figure 1c; shows log_2_ expression). The ratio of cytotoxic CD8^+^ T cells by FOXP3^+^ regulatory T cells has been shown to have prognostic and predictive value in multiple cancer types [40,41,42]. We assessed this ratio using *CD8A* and *FOXP3* gene expression and found greater than 3-fold upregulation in patient 1 at both T2 and T3 compared to baseline. This ratio, however, was lower in patient 2 after RFA treatment (Figure 1d).

Further, genes associated with proteins that participate in antigen presentation to T cells via major histocompatibility complexes I and II (*CD1E* for MHC-I [43,44] and *HLADQA1* for MHC-II [45]) were also highly upregulated upon RFA. There was a 4.5-fold and 5.3-fold increase in expression of *CD1E* and *HLADQA1*, respectively, at T3 time point in patient 1. *CD1E* expression in patient 2 at T3 was also found to be 3.9-fold greater than at baseline, but a decreasing trend in *HLADQA1* was observed (Figure 1e). However, we warrant further validation of MHC-I and -II using additional gene sets representing the complexes in the future.

Next, we investigated the different immune cell types in tumours pre- and post-RFA. Patient 1 showed RFA course-dependent upregulation in T cell markers–*CD4* (8.7-fold at T3) and *CD8* (35.7-fold at T3), along with a 3.7-fold increase in markers representing NK cells. Genes representing macrophages and mast cells showed modest change in patient 1 with respect to RFA treatment. *CD4* and *CD8* expression showed a 2-fold and 3.2-fold decrease at T3 compared to baseline in patient 2 (Figure 1f). The baseline levels of CD4 and CD8 were, however, higher in patient 2 compared to patient 1.

A reduction in transcript levels of genes representing macrophages (2.6-fold) and mast cells (3.7-fold) and no change in NK cell genes were observed in patient 2 post-RFA (at T3) compared to baseline (Figure 1f). Further, apart from upregulated T cell markers, 6.4-fold and 8-fold increases in cytotoxic T cell function score and inflammation score, respectively, were observed at T3 compared to baseline in patient 1. A decrease in cytolytic function score (14.17-fold at T3) and a 2-fold increase in inflammation score were observed after two courses of RFA in patient 2 (Figure 1g).

We studied the expression of known immune checkpoint genes with agonists/antagonists currently in clinical trials or approved for clinical use in other cancers [46,47,48]. After 2 courses of RFA (T3), patient 1 showed a >2-fold increase in *CD274 (PDL1)*, *IDO1*, *TNFRSF18* (*GITR)*, Figure 1h. In addition, a >2-fold upregulation in *PDCD1* (*PD1)*, *IDO1*, *TNFSF18 (GITRL)* and *TNFRSF18 (GITR)* was observed in patient 2 at T3, shown in Figure 1i.

### 3.2. Feasibility of using FNABs to Evaluate the Tumour Stroma in RFA-Treated PDACs

**Figure 2:**
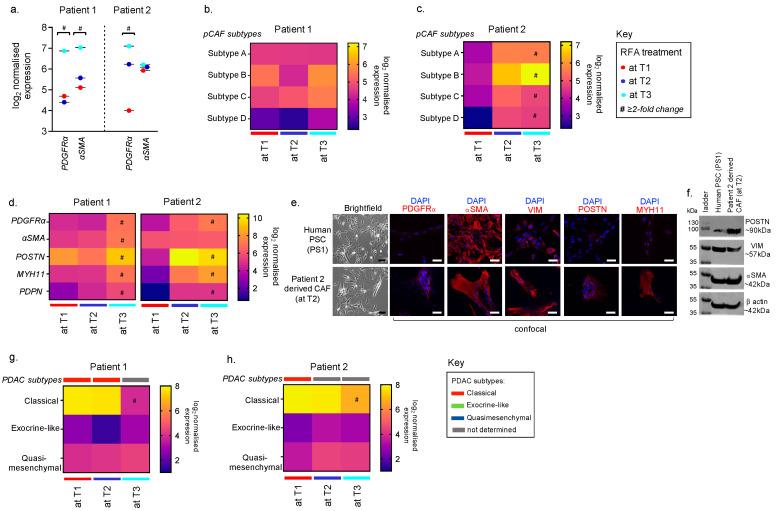
Longitudinal FNAB from RFA-treated patients and TME. (**a**) Dot plot showing upregulation of pan-CAF markers-*PDGFRα* and *ACTA2* (*αSMA*) as a response to RFA in patient 1 and 2, respectively. (**b**,**c**) Heatmaps representing a switch in the CAF subtypes over the course of RFA in patient 1 and 2, respectively; separate scale bars for the two patients depicting averaged log_2_ normalised subtype-specific CAF gene expression. (**d**) Heatmap representing change in IHC validated exclusive pCAF subtype-specific genes over the course of RFA in patient 1 and 2. (**e**) Brightfield (10× magnification on Leica DMi8; scale bar represents 100 μm) and confocal (20× magnification on Zeiss LSM700; scale bar represents 50 μm) images of different CAF markers in patient 2 T2-derived CAFs and normal (immortalised) human pancreatic stellate cells, PS1. (**f**) Western blot showing expression of different CAF markers in patient 2 T2-derived CAFs and normal (immortalised) human pancreatic stellate cells, PS1. (**g**,**h**) Heatmaps representing a switch in the PDAC subtypes over the course of RFA in patient 1 and 2, respectively; colour bars represent NTP-derived PDAC subtype and separate scale bars for the two patients depicting averaged log_2_ normalised gene expression associated with each PDAC subtype. # represents greater than or equal to two-fold change in gene expression between T1 and T3.

Recent studies have reported loco-regional coagulation and necrosis as a result of RFA resulting in a remodelling of the TME [10,49]. Data from both patients at T3 indicated a marked upregulation (4.5-fold; patient 1 and 8.5-fold; patient 2, respectively) of pan-CAF marker-*PDGFRα*. Patient 1’s tumour also showed a 3.8-fold increase in *ACTA2* (*αSMA*) after 2 courses of RFA, Figure 2a. RFA led to a modest increase in subtype B and C CAFs in patient 1, Figure 2b. In contrast, the RFA-treated patient 2 tumour (T3) showed a 3.6-fold, 9-fold and 5-fold upregulation of pCAF subtypes A, B and D, respectively, compared to baseline (Figure 2c). We then selected the genes that have been previously validated by immunohistochemistry to exclusively represent pCAF subtypes and found an enrichment of *POSTN* (subtype A), *MYH11* (subtype B), and *PDPN* (subtype C) in both patients after RFA; however, the increase was pronounced particularly in the patient 2 tumour at T2 and T3, Figure 2d. We attempted to establish CAF cultures from fresh patient FNABs and successfully obtained CAFs from the patient 2 tumour at the T2 timepoint. An increased expression of pan-CAF markers-PDGFRα, αSMA and VIM was observed along with an expression of subtype A (POSTN) and subtype B (MYH11) in the cultured CAFs in comparison to the normal (immortalised) human pancreatic stellate cell line, PS1 [18,50,51] (Figure 2e,f; Table S2; Supplementary Methods). The expression of these markers in cultured CAFs may suggest CAF-specific expression of these markers in the patient 2 FNAB at T2.

### 3.3. Feasibility of Using FNABs to Study Cancer Subtypes in RFA-Treated PDACs

Our data indicated that both patients at baseline were categorised into the classical PDAC subtype. RFA led to a marked decrease in genes representing the classical PDAC subtype in both patients at T3 compared to baseline and T2 (Figure 2g,h). No particular change in the exocrine-like or quasi-mesenchymal PDAC subtype was observed in either of the patients upon RFA.

## 4. Conclusions

This feasibility study established the value of longitudinal sampling with EUS-FNABs to study local TME changes occurring in response to RFA in two patients. We successfully isolated RNA and cultured CAFs from these biopsies to comprehensively profile immune, CAF and cancer cell gene expression using a custom panel of genes associated with PDAC subtypes (both cancer and CAF) and immune cell types. In this case study, we have attempted to elucidate immune modulation in response to RFA in two patients depending on their baseline immune repertoire. This study in PDAC paves the way to access samples longitudinally during treatment in clinical trials with EUS-RFA to assess the molecular changes and subtypes associated with responses to therapy.

Despite the limitations of these data from two patients and only 144 genes, this is the first feasibility study to comprehensively report a transcriptomic profile of the TME during RFA treatment by longitudinal sampling using serial EUS-FNABs in PDAC patients. Hence, this warrants further study with an increased sample size for a comprehensive study using EUS-RFA-based molecular changes in PDAC.

## Data Availability

The data presented in this study are available on request from the corresponding authors.

## Supplementary Materials

The following supporting information can be downloaded at: https://www.mdpi.com/article/10.3390/curroncol29100531/s1, Figure S1: Mycoplasma screening by polymerase chain reaction for patient 2-derived CAFs at T2 (after 1st RFA). Agarose gel electrophoresis image representing mycoplasma negative CAFs assessed by both initial and nested PCR. β-actin (ACTB) gene was used as a reference control; Figure S2: DNA STR profiling of patient 2-derived CAFs at T2 (after 1st RFA) by polymerase chain reaction. 16 independent loci were investigated by AmpFISTR® Identifier® Plus PCR amplification kit and analysed; Table S1: Derivation of gene expression scores used to estimate immune, CAF and cancer cell subtypes or/and function; Table S2: List of antibodies used.
