# Supplementary material for: A Case Report on Longitudinal Collection of Tumour Biopsies for Gene Expression-Based Tumour Microenvironment Analysis from Pancreatic Cancer Patients Treated with Endoscopic Ultrasound Guided Radiofrequency Ablation
Source: Curr Oncol. 2022 Sep 21;29(10):6754–63. doi: 10.3390/curroncol29100531 (PMC9600136; doi:10.3390/curroncol29100531)
Supplement: Supplementary file 1 [file curroncol-29-00531-s001.zip › curroncol-1858425-supplementary.pdf]

**A case report on longitudinal collection of tumour biopsies for gene expression-based tumour microenvironment analysis from pancreatic cancer patients treated with endoscopic ultrasound guided radiofrequency ablation**

## Supplementary Information

## Supplementary Methods

*Isolation of patient-derived cancer-associated fibroblasts from EUS-FNABs*

Cancer associated fibroblasts from fresh FNABs were isolated by outgrowth method[1,2]. Briefly, tissue biopsies were transported in advanced Dulbecco's Modified Eagle Medium/Ham's F12 (Gibco, NY, USA) containing 10% fetal bovine serum (FBS, Qualified HI; Gibco, NY, USA) and 1% penicillin-streptomycin solution (Gibco, NY, USA) (complete media). FNABs were washed twice in phosphate buffered saline (PBS) containing 1% penicillin-streptomycin solution and cut into smaller pieces using sterile scalpels. Tissue digestion was performed in PBS containing 1mM ethylenediamine-tetraacetic acid (PBS-EDTA) along with 2x TrypLE (Gibco, NY, USA) and 20 µl of DNase enzyme for 1 h at 37°C. Following digestion, tubes were centrifuged for 5 min at 1200 rpm and the tissue pellets were re-suspended in 2 ml of ACK lysis buffer (Gibco, NY, USA) to deplete red blood cells by incubating on ice for 3 min. 5 ml of complete media was added to neutralize the solution and tubes were centrifuged for 5 min at 1200 rpm. 3 ml of pre-warmed complete media was added to the tissue pellet and contents were placed on 35 mm dishes. After a week, fibroblasts that grew out from the tissue bits were trypsinised and passaged once a week. Cells were maintained at 37°C in a 5% $CO_2$ incubator.

*Immunofluorescence*

$2 \times 10^4$ patient-derived CAFs or PS1 cells (normal immortalised human pancreatic stellate cells)[3] were seeded on 8-well chambered coverslips (ibidi, Thistle Scientific, Glasgow, UK) in complete growth media. Next day, media was removed by aspiration and cells were washed twice with cold PBS and fixed in 4% paraformaldehyde for 15 min at room temperature (RT). After two gentle washes in PBS, cell permeabilisation and blocking was performed using PBS containing 5% bovine serum albumin (BSA) + 0.3% Triton-X-100 for 1 h at RT. Cells were incubated with primary antibodies overnight at 4°C. On the following day, cells were washed thrice with PBS and incubated with secondary antibody (in PBS containing 1% BSA + 0.05% Tween-20) for 1 h at RT in a moist dark chamber. Cells were washed thrice with PBS and

stained with 4',6-diamidino-2-phenylindole (DAPI) (1:1000 in PBS) for 10 min at RT. Coverslips were mounted on 50 μl of VectaShield mounting media (Vector Labs, Detroit, USA) and imaged using Zeiss LSM700 (Jena, Germany). Antibody information is listed in Table S2.

*Immunoblotting*

0.6 x $10^6$ patient derived CAFs or PS1 cells (normal immortalised human pancreatic stellate cells)[3] were seeded in complete growth media in 60 mm dishes. Next day, media was aspirated, and cells were washed twice with cold PBS. 30 μl of cold NP40 lysis buffer (50mM Tris-HCL, 150mM sodium chloride, 1% NP40) containing phosphatase and protease inhibitors (Pierce, Life Technologies Corporation, CA, USA) was added to obtain protein lysates. Protein concentrations were determined using BCA method (Pierce, Life Technologies Corporation, CA, USA) and 10 μg of protein was fractionated onto 12% sodium dodecyl sulphate-polyacrylamide gels. Blotting was performed on polyvinylidene difluoride (PVDF) membranes for 1 h at 100 V. Membranes were blocked in PBS containing 5% BSA + 0.12% Tween-20 for 1 h followed by probing with primary antibodies, overnight at 4°C. Next day, membranes were washed and incubated with anti-mouse/rabbit secondary antibodies conjugated to horseradish peroxidase. Immunoreactive bands were visualised on Licor Odyssey Fc (Cambridge, UK) using enhanced chemiluminescence (ECL, Merck Millipore, Darmstadt, Germany). β-actin was used a loading control. Antibody information is listed in Table S2.

**Table Legends**

Table S1. Derivation of gene expression scores used to estimate immune, CAF and cancer cell subtypes or/and function.

Table S2. List of antibodies used.

**Figure Legends**

Figure S1. Mycoplasma screening by polymerase chain reaction for patient 2-derived CAFs at T2 (after 1st RFA). Agarose gel electrophoresis image representing mycoplasma negative CAFs assessed by both initial and nested PCR. β-actin (*ACTB*) gene was used as a reference control.

Figure S2. DNA STR profiling of patient 2-derived CAFs at T2 (after 1st RFA) by polymerase chain reaction. 16 independent loci were investigated by AmpFISTR® Identifier® Plus PCR amplification kit and analysed.

**References**


1. Apte M V, Haber PS, Applegate TL, et al. Periacinar stellate shaped cells in rat pancreas: identification, isolation, and culture. *Gut*. 1998;43(1):128-133. doi:10.1136/gut.43.1.128

2. Bachem MG, Schneider E, Gross H, et al. Identification, culture, and characterization of pancreatic stellate cells in rats and humans. *Gastroenterology*. 1998;115(2):421-432. doi:10.1016/s0016-5085(98)70209-4

3. Li NF, Kocher HM, Salako MA, Obermueller E, Sandle J, Balkwill F. A novel function of colony-stimulating factor 1 receptor in hTERT immortalization of human epithelial cells. *Oncogene*. 2009;28(5):773-780. doi:10.1038/onc.2008.412

# Figure S1

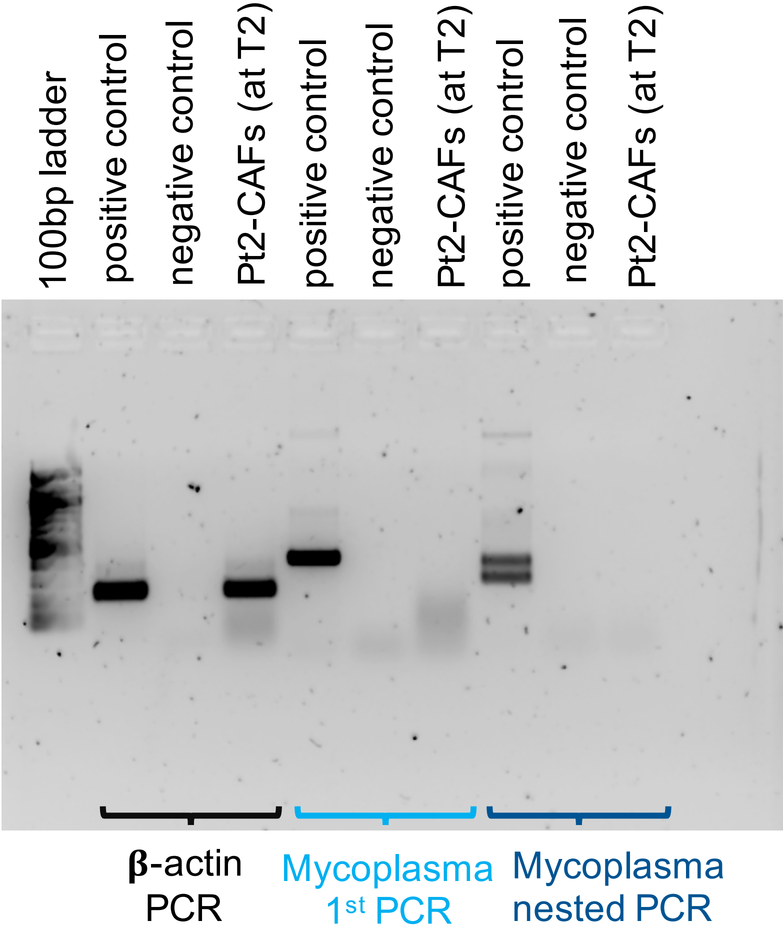
100bp ladder
positive control
negative control
Pt2-CAFs (at T2)
positive control
negative control
Pt2-CAFs (at T2)
positive control
negative control
Pt2-CAFs (at T2)
β-actin PCR
Mycoplasma 1st PCR
Mycoplasma nested PCR

Figure S2

Eurofins Genomics Europe Applied Genomics GmbH, Anzinger Str. 7 a, D-85560 Ebersberg

SOP_APG_Zelllinienauthentizität_A04_1.0

**Certificate**

Report date: 30.06.2021

**Method:** Genetic characteristics were determined by PCR-single-locus-technology. 16 independent PCR-systems D8S1179, D21S11, D7S820, CSF1PO, D3S1358, TH01, D13S317, D16S539, D2S1338, AMEL, D5S818, FGA, D19S433, vWA, TPOX and D18S51 were investigated. (ASN-0002 core markers are colored grey, Thermo Fisher, AmpFlSTR® Identifiler®Plus PCR Amplification Kit) In parallel, positive and negative controls were carried out yielding correct results.

**Result:**

| **Sample Name** | ARDEO Pt2 CAF at T2 |
|---|---|
| D8S1179 | 15,16 |
| D21S11 | 30,32.2 |
| D7S820 | 10,11 |
| CSF1PO | 11,12 |
| D3S1358 | 15,17 |
| TH01 | 6,9 |
| D13S317 | 11,12 |
| D16S539 | 9,11 |
| D2S1338 | 21,25 |
| D19S433 | 13,13 |
| vWA | 16,17 |
| TPOX | 9,9 |
| D18S51 | 12,16 |
| AMEL | X,Y |
| D5S818 | 8,9 |
| FGA | 19,21 |

The table shows the result of the cell line analysis. Please note that only the PCR-systems according to ANSI/ATCC standard ASN-0002 were aligned (D5S818, D13S317, D7S820, D16S539, VWA, TH01, TPOX, CSF1PO, AMEL - coloured grey).

**Table S1. Derivation of gene expression scores used to estimate immune cells/function, cancer and cancer-associated fibroblast subtypes.**

| Scores | Genes |
|---|---|
| **Immune** | |
| T cell score | *CCL19, CCR5, CD2, CD3G, CD4, CD8, FOXP3, ITK, LRRN3, LY9, SPN, TAL1, CD37, CXCL13* |
| B cell score | *BLK, CD19, CD22, FAIM3, FCER2, MS4A1, PAX5, POU2F2* |
| Cytolytic function score | *GZMA, GZMK* |
| Inflammation score | *CXCL9, IFIT3, IFNG, STAT1* |
| MHC-I | *CD1E* |
| MHC-II | *HLA-DQA1* |
| | |
| **Cancer (PDAC Subtypes)** | |
| Classical | *AGR2, ATP10B, CAPN8, CEACAM5, CEACAM6, ELF3, ERBB3, FOXQ1, FXYD3, GPRC5A, GPX2, LGALS4, MUC13, PLS1, S100P, SDR16C5, ST6GALNAC1, TFF1, TFF3, TMEM45B, TOX3, TSPAN8* |
| Exocrine-like | *CEL, CELA2B, CELA3A, CELA3B, CFTR, CLPS, CPB1, CTRB2, GP2, PLA2G1B, PNLIP, PNLIPRP2, PRSS1, PRSS2, REG1A, REG1B, REG3A, SLC3A1, SLC4A4, SPINK1* |
| Quasimesenchymal | *AHNAK2, AIM2, CAV1, CKS2, FAM26F, FERMT1, GPM6B, HK2, HMMR, KRT14, LOX, NT5E, PAPPA, PHLDA1, PMAIP1, S100A2, SLC16A1, SLC2A3, SLC5A3, TWIST1* |
| | |
| **CAFs (pCAF Subtypes)** | |
| Subtype A | *ANPEP, ENPP2, PFKFB1, POSTN, PRR15L, TNC* |
| Subtype B | *CDH1, COMP, IL1RL1, ITM2A, MYH11, SFRP2* |
| Subtype C | *ANGPTL2, HPSE, OAS1, PDPN, RGCC, SEMA3E, TNFSF10* |
| Subtype D | *ACVR1C, AQP1, GPR56, MEOX2, PRELP* |

**Table S2. List of antibodies used for characterisation of patient-2-derived CAFs.**

| Antibody | Supplier | Dilution | Application |
|---|---|---|---|
| **Primary antibodies** | | | |
| PDGFR$\alpha$ | Abcam (ab203491) | 1:100 | Immunofluorescence |
| $\alpha$SMA | Abcam (ab124964) | 1:250/1:1000 | Immunofluorescence/Immunoblotting |
| Vimentin | CST (CST5741) | 1:100/1:1000 | Immunofluorescence/Immunoblotting |
| POSTN | Abcam (ab215199) | 1:100/1:1000 | Immunofluorescence/Immunoblotting |
| MYH11 | Abcam (ab683) | 1:100 | Immunofluorescence |
| Beta-actin | Sigma (A1978) | 1:5000 | Immunoblotting |
| **Secondary antibodies** | | | |
| Alexa Fluor 568 donkey anti-rabbit IgG (H+L) | Life Technologies (A10042) | 1:500 | Immunofluorescence |
| Goat anti-mouse Immunoglobulins HRP | DAKO (P0447) | 1:5000 | Immunoblotting |
| Goat anti-rabbit Immunoglobulins HRP | DAKO (P0448) | 1:5000 | Immunoblotting |
